# Association of iron overload based quantitative T2* MRI technique and carotid intima-media thickness in patients with beta-thalassemia: A cross-sectional study

**DOI:** 10.1186/1471-2261-10-62

**Published:** 2010-12-31

**Authors:** Shahram Akhlaghpoor, Morteza Hoseini, Amirhosein Jafarisepehr

**Affiliations:** 1 Noor Medical Imaging Center, Tehran, Iran; 2 Department of Radiology, Tehran University of Medical Sciences, Sina Hospital, Tehran, Iran

## Abstract

**Background:**

Body iron status has been implicated in atherosclerotic cardiovascular disease. The main hypothesis is that high iron status is associated with increased risk of atherosclerosis. We investigated the potential role of iron as an additional risk factor promoting atherosclerosis among beta-thalassemic patients.

**Methods:**

In this cross-sectional study, the liver iron load was assessed by quantitative T2* MRI technique and intima-media thickness (IMT) of the common carotid artery by high-resolution ultrasound among 119 patients (62 male, 57 female) with beta-thalassemia (major and intermediate) whose age ranged from 10 to 50 years with a mean of 25.6 years. The patients were divided into three groups according to the severity of iron loading, obtained by T2*MRI technique: group I (normal), group II (mild) and group III (moderate and severe) iron load.

For elimination of the effect of age on carotid IMT values, the patients also were divided into four age groups (10-19 y, 20-29 y, 30-39 y and 40-50 y). Mean carotid IMT based on the severity of iron loading were compared at different age groups, using one way ANOVA analysis for assessing the effect of iron loading on carotid IMT. Pearson's coefficient of correlation were used to assess the degree of correlation between studied variables (liver T2*, IMT, age).

**Results:**

There were significant differences in mean carotid IMT based on the severity of iron loading at different age groups, with P = 0.003 at 20-29 y, P = 0.006 at 30-39 y and p = 0.037 at 40-50 y. Age (p = 0.001) and liver T2*(p = 0.003) had significant correlation with mean carotid IMT independently.

At the age group of 10-19 years, there were not significant differences in mean carotid IMT based on the liver iron loading (p = 0.661).

No significant differences also are seen in mean carotid IMT between male and female (p = 0.41).

**Conclusions:**

This study identified a relationship between body iron status and carotid IMT. This relationship support to the hypothesis of a link between body iron load and atherosclerosis.

## Background

Thalassemia is considered the most common genetic disorder worldwide[1]. It is result from a defect in the synthesis of one or more of the subunits of hemoglobin.

In b-thalassemia, the b-chains of hemoglobin have a normal structure but are produced in reduced or undetectable amounts, resulting in excess of α-chains, which are unstable and precipitate to form intracellular inclusion bodies. This excessive intracellular deposition of α-chain material is responsible for peripheral hemolysis of the erythrocytes [2-4]. Conventional treatment of the patients with b-thalassemia major is repeated blood transfusions.

Repeated blood transfusions and peripheral hemolysis leads to iron overload initially in reticulo-endothelial system and secondary to all parenchymal organs, mainly heart, pancreas, pituitary gland, and gonads, with cytotoxic effects [5].

Accumulation of iron in excess of physiologic requirements has been implicated in risk of cardiovascular disease, because of the catalytic role of iron in free radical reactions, cause oxidative stresses on vessel wall [6-8].

Increased common carotid IMT is a marker of early atherosclerosis and has been associated with cardiovascular risk and risk of coronary artery disease events [9,10].

First, Sullivan in 1981 formulated the iron-heart hypothesis of atherosclerotic cardiovascular disease. According to this hypothesis, the loss of iron with menstruation explains the lower risk of coronary heart disease(CHD) in premenopausal women compared with men and postmenopausal women [11].

This, accompanied by the observation that hysterectomy without oophorectomy was associated with an increased CHD risk [12,13].

Strong epidemiological evidence is available that iron is an important factor in the process of atherosclerosis. But some studies do not support the role of iron as a potent risk factor in CHD and the role of iron in promotion of atherogenesis remains controversial [13,14].

An explanation for these discrepant results may lie in the limitations of the different markers that were used for iron load assessing, for example, serum iron and transferrin saturation are affected by inflammation and diurnal variation and serum ferritin levels is affected by factors, such as chronic inflammatory and Infectious disease, hematological and other malignancies and liver dysfunction [5,15].

Therefore, it is quite possible that the levels of serum ferritin are influenced by inflammation independently of iron stores [16,17].

This may explain the failure of some previous studies find an association between ferritin and CHD.

The liver iron concentration (LIC) is a reliable indicator of total body iron stores in thalassemic patients [18]. MRI measures the LIC indirectly by detecting the paramagnetic effect produced by iron deposition. Deposition of iron in the liver increases the magnetic field heterogeneities, resulting in a decreased T2* relaxation value of the liver that leads to a decline in liver MRI signal, proportionally to the amount of the LIC [19,20].

This method (T2*MRI) is a quantitative, non-invasive, accurate, widely available and reproducible method for estimating iron over load in different tissues [21,22].

Therefore, in this study we were used from quantitative T2*MRI technique, for estimation of the liver iron stores (as the indicator of body iron load).

The purpose of this study was to evaluate the role of iron as a potent risk factor in early atherosclerosis.

## Methods

### Subjects

In this cross-sectional study, we enrolled 119 patients (62 male, 57 female) with beta -thalassemia major and intermediate that referred from the hematology clinic for routine assessment of the liver iron load by MRI imaging.

The patient's age ranged from 10 to 50 years with a mean of 25.6 years.

All of the patients had been received chelation therapy with subcutaneous deferoxamine. Smokers and patients with heart failure, previous history of cardiovascular event, systemic hypertension, diabetes mellitus, hyperlipidemia, and thyroid dysfunction were excluded, to eliminate the effect of these factors on carotid IMT.

The patients based on the compliance, to chelation therapy had different liver iron load.

Liver iron load was determined by quantitative T2* MRI technique and the patients based on the severity of iron loading were divided into three groups: group I (normal), group II (mild) and group III (moderate and severe) iron load. Severity of iron loading is defined as follows:

MRI T2* values greater than 6.3 ms was categorized as normal (T2* > 6.3 ms), mild (2.8-6.3 ms), moderate (1.4-2.7 ms) and severe (T2* < 1.4 ms) [23,24].

Mean carotid IMT were assessed in the right and left common carotid arteries by high-resolution ultrasound in the mentioned groups. To elimination of the effect of age in carotid IMT values, the patients also were divided into four age groups. (10-19 y, 20-29 y, 30-39 y and 40-50 y)

Then mean carotid IMT values based on the liver iron loading were compared at different age groups.

The Institutional Ethics Committee approved the study and all subjects gave informed consent.

### MRI protocol

MRI scans were performed with a 1.5 T superconducting MR imager (Siemens Symphony Imager, Erlangen, Germany), using the method described by Anderson et al [21].

A standard quadrature RF body coil was used in all measurements for both excitation and signal detection.

All subjects were placed in a supine position and entered the magnet cradle, using the head-first configuration. Respiratory triggering was used to monitor the patients' breathing. Spatial presaturation slabs were used to suppress motion-related artifacts. The MRI T2* of the liver was determined using a single 10 mm slice through the center of the liver scanned at 12 different echo times (TE 1.3-23 ms).

Each image was acquired during a 11-13 s breath-hold using a gradient-echo sequence (repetition time 200 ms, flip angle 20◦, base resolution matrix 128 pixels, field of view 39.7 × 19.7 cm, sampling bandwidth of 125 kHz).

The signal intensity of the liver parenchyma and the background noise were measured in each of the 12 images, using in-house software (CMR tools, Imperial College). Background noise was subtracted from the liver signal intensity, and the net value was plotted against the echo time for each image. A trend line was fitted to the resulting exponential decay curve, with an equation of the form y = Ke^TE/T2^* where K represents a constant, TE represents the echo time and y represents the image signal intensity.

### Carotid ultrasound

Longitudinal B-mode scans of the common carotid artery were obtained, using ALOKA ultrasound system (model prosound α5 sx, company Ltd, Japan) with 10-MHZ linear array transducer. The gain was set at 60-70 dB that was usual in clinical practice. The far wall was assessed just 2 cm proximal to the carotid bifurcation, to identify the maximal IMT, by manually.

IMT defined as the distance between the junction of the lumen and intima and that of the media and adventitia [10,25].

Three measurements were made in the right and left common carotid arteries and were averaged to determine the IMT for each side.

### Statistical analyses

Summary of data are presented as mean values ± one standard deviation.

The correlations between parameters (liver T2*, IMT, age) were analyzed, using Pearson's coefficient of correlation. To compare quantitative variables with different groups, one way ANOVA analysis were used.

kruskal-wallis analysis also were used, due to relatively low sample size in this study.

All statistical analyses were assessed using SPSS software for windows ver.18 (SPSS Inc., Chicago, IL, USA). A p-value less than 0.05 was considered statistically significant.

## Results

Table 1 shows summary of general characteristics of the patients including age, weight, height, age at diagnosis, transfusion start age, transfusion interval, transfusion volume, deferoxamine start age and mean duration of Chelation therapy.

**Table 1:** General characteristics of the patients

Parameter	Mean	SD*
Age (year)	25.6	± 9.2
BMI	20.8	± 2.9
Age at diagnosis (month)	53	± 79.1
Transfusion start age (month)	44	± 66.1
Transfusion volume (ml)	807	± 216
Transfusion interval (day)	29	± 24.8
Deferoxamine start age (month)	104	± 115
Duration of deferoxamine treatment (year)	17	± 6.3

*SD: standard deviation

Table 2 shows Pearson's linear correlation coefficients for simple regression analysis of the carotid IMT with the other parameters (age and liver T2*). Age (as a continuous variable) and liver T2* had significant correlation with mean carotid IMT independently.

**Table 2:** Pearson's correlation coefficients between the IMT and parameters (age, Liver T2*)

	Pearson's correlation P-value	P-value
**Age**	0.68	0.001
Liver T2*	0.26	0.003

**IMT**: dependent variable **age**, Liver T2*: independent variables. P-value < 0.05 is significant.

Table 3 shows Mean carotid IMT based on the severity of liver iron loading at different age groups. At the age group of 10-19 years, no significant differences were found in mean carotid IMT based on the iron loading.

**Table 3:** Mean carotid IMT based on the severity of iron loading at different age groups.

Range of age (year)	Iron Load	Mean IMT	N*	SD"	P-value
**10-19**	Normal	0.49	6	0.02	
	Mild	0.51	7	0.06	0.661
	Moderate & Severe	0.51	18	0.04	

**20-29**	Normal	0.50	15	0.05	
	Mild	0.57	17	0.07	0.003
	Moderate & severe	0.59	27	0.08	

**30-39**	Normal	0.53	4	0.04	
	Mild	0.64	5	0.05	0.006
	Moderate & Sever	0.76	6	0.12	

**40-50**	Normal	0.63	3	0.05	
	Mild	0.77	5	0.08	0.037
	Moderate & Severe	0.90	6	0.17	

*N: number of patients. "SD: standard deviation

At the other age groups, mean carotid IMT in group III (moderate and severe) was higher than group II (mild) and in group II (mild) was higher than group I(normal). Differences in mean carotid IMT were significant at these age groups based on the iron loading.

With kruskal-wallis analysis, the result was the same and differences in mean carotid IMT were significant in all age groups with exception of 10-19 year; with p = 0.5 at 10-19 y, p = 0.004 at 20-29 y, p = 0.006 at 30-39 y and p = 0.021 at 40-50 y.

**Figure 1:**
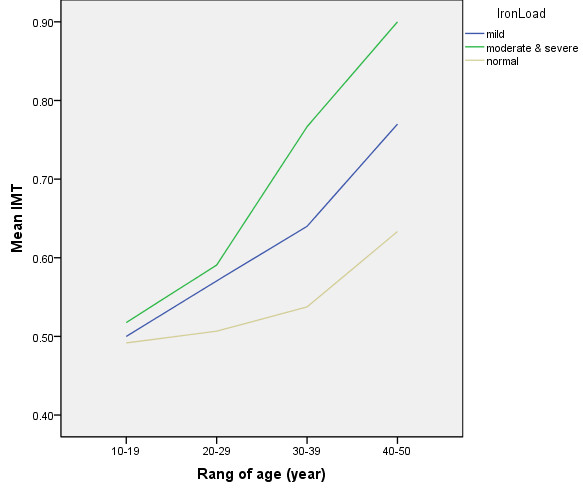
**Line chart of the mean carotid IMT based on the severity of iron load**.

Figure 1 shows Line chart of the differences in mean carotid IMT based on the severity of iron loading at different age groups. These differences are lower in the younger patients and increase with relatively exponential curve in the older patients.

No significant differences are seen in mean carotid IMT between male and female. (p = 0.41)

## Discussion

Carotid IMT is considered an early marker of atherosclerotic process and is currently used to assess the presence and the progression of atherosclerosis [9,10]. This study finds an association between iron overload and early atherosclerosis as reflected by increased carotid IMT in the thalassemic patients. Our data support the hypothesis that iron is a risk factor of CHD. Epidemiological studies examining the role of iron in cardiovascular disease have yielded conflicting results [9,26]. These conflicting results may due to the use of different indicators of iron stores and their modulation by various factors[9].

Some investigators have hypothesized that iron may be primarily involved in the early events of atherosclerosis, and focusing on cardiovascular morbidity and mortality (reflecting later stage of the disease) may not give insight in to the potential mechanistic role of iron [12,27].

Mentioned reasons explain the failure of some previous studies to finding an association between body iron load and CHD.

At the age group of 10-19 years, no significant differences are seen in mean carotid IMT based on the severity of iron loading. This finding could be resulting from this fact that, atherogenesis is a prolonged process[28] and should not expect to present at early young patients.

In healthy person's, carotid IMT is significantly higher in male in comparison to female [29]. At present study there were not significant differences in mean carotid IMT between male and female, which is in agreement with iron hypothesis. Because in thalassemic patients, body iron load is in the same ranges in male and female. On opposite, in healthy person's body iron load is significantly higher in male in comparison to female.

The mechanism by which iron may stimulate atherogenesis is unclear.

It is suggested that the catalytic role of iron in free radical reactions cause oxidation of LDL and may be an important factor in the formation of atherosclerotic lesions. Studies have shown that iron can stimulate lipid peroxidation in vitro and in vivo [13].

Iron-catalyzed free radical reactions cause oxidation of LDL, which occurs in endothelial cells, smooth muscle cells, lymphocytes, or macrophages.

Normal LDL can cross the arterial wall without causing damage to the vessel wall. Unlike normal LDL, oxidized LDL is recognized by macrophages, followed by accumulation of lipids in these cells and the formation of foam cells, the characteristic cells of the fatty-streak lesions of early Atherosclerosis. Oxidized LDL also has chemotactic activity that provides recruitment of circulating monocytes to the vessel wall and prevents the exiting of macrophages from the intima of the arterial wall.

Thereby, oxidized LDL has cytotoxic capacity that induces changes in endothelial cells with loss of endothelial integrity, which could facilitate further accumulation of both circulating monocytes and LDL and thus promote the progression of the atherosclerotic lesion [13,30-33].

Thalassemic patients have mostly blood lipid levels within the normal range and Prevalence of lipid and lipoprotein abnormalities is much lower as compared to the general population of the same age[34], but carotid IMT is significantly higher in thalassemic patients in comparison with the healthy persons[35]. This subject shows the important role of the body iron store in atherosclerosis process.

The clinical importance of this study is prevention from the progression of atherosclerosis in early stages by decrease in body iron load.

Decrease in body iron load can achieve by good compliance of chelation therapy in the thalassemic patients and by blood donation in healthy persons.

A potential limitation of this study is the cross-sectional and observational nature of our study. This type of study cannot identify a cause-and-effect relationship, but associations can be examine. another limitation is the relative low sample size, especially at the high age groups.

Findings from the current study may be applied to thalassemic patients, but cannot be extrapolated directly to the general population.

## Conclusions

In summary, we observed an independent relationship between body iron load and carotid atherosclerosis. Our findings are in agreement with previous results and Support to the hypothesis that iron is linked to cardiovascular disease.

## Abbreviations

IMT: intima-media thickness; CHD: coronary heart disease; LIC: liver iron concentration; LDL: Low density lipoprotein; T2* MRI: T2-star Magnetic resonance imaging

## Competing interests

The authors declare that they have no competing interests.

## Authors' contributions

**MH** carried out the measurement and acquisition of the MRI and IMT data. **AJS **conceived of the study, and participated in the design of the study and performed the statistical analysis. **SA **participated in the design of study and coordination and helped to draft the manuscript. All authors read and approved the final manuscript.

## Pre-publication history

The pre-publication history for this paper can be accessed here:

http://www.biomedcentral.com/1471-2261/10/62/prepub

## References

[B1] Ruiz-Arguelles GJ Lopez-Martinez B Ruiz-Reyes G Heterozygous beta-thalassemia: not infrequent in Mexico Arch Med Res 2001 32 4 293 295 10.1016/S0188-4409(01)00284-3 11440786

[B2] Aldouri MA Wonke B Hoffbrand AV Flynn DM Laulicht M Fenton LA Scheuer PJ Kibbler CC Allwood CA Brown D Iron state and hepatic disease in patients with thalassaemia major, treated with long term subcutaneous desferrioxamine J Clin Pathol 1987 40 11 1353 1359 10.1136/jcp.40.11.1353 3121679 PMC1141239

[B3] Mavrogeni SI Maris T Gouliamos A Vlahos L Kremastinos DT Myocardial iron deposition in beta-thalassemia studied by magnetic resonance imaging Int J Card Imaging 1998 14 2 117 122 10.1023/A:1005922016048 9617642

[B4] Argyropoulou MI Astrakas L MRI evaluation of tissue iron burden in patients with beta-thalassaemia major Pediatr Radiol 2007 37 12 1191 1200 quiz 1308-1199 10.1007/s00247-007-0567-1 17710390 PMC2292491

[B5] Christoforidis A Haritandi A Tsitouridis I Tsatra I Tsantali H Karyda S Dimitriadis AS Athanassiou-Metaxa M Correlative study of iron accumulation in liver, myocardium, and pituitary assessed with MRI in young thalassemic patients J Pediatr Hematol Oncol 2006 28 5 311 315 10.1097/01.mph.0000212915.22265.3b 16772883

[B6] Papanikolaou G Pantopoulos K Iron metabolism and toxicity Toxicol Appl Pharmacol 2005 202 2 199 211 10.1016/j.taap.2004.06.021 15629195

[B7] Qayyum R Schulman P Iron and atherosclerosis Clin Cardiol 2005 28 3 119 122 15813617

[B8] Shah SV Alam MG Role of iron in atherosclerosis Am J Kidney Dis 2003 41 3 Suppl 1 S80 83 10.1053/ajkd.2003.50091 12612959

[B9] Drueke T Witko-Sarsat V Massy Z Descamps-Latscha B Guerin AP Marchais SJ Gausson V London GM Iron therapy, advanced oxidation protein products, and carotid artery intima-media thickness in end-stage renal disease Circulation 2002 106 17 2212 2217 10.1161/01.CIR.0000035250.66458.67 12390950

[B10] Gaenzer H Marschang P Sturm W Neumayr G Vogel W Patsch J Weiss G Association between increased iron stores and impaired endothelial function in patients with hereditary hemochromatosis J Am Coll Cardiol 2002 40 12 2189 2194 10.1016/S0735-1097(02)02611-6 12505233

[B11] Sullivan JL Iron and the sex difference in heart disease risk Lancet 1981 1 8233 1293 1294 10.1016/S0140-6736(81)92463-6 6112609

[B12] Ferrara DE Taylor WR Iron chelation and vascular function: in search of the mechanisms Arterioscler Thromb Vasc Biol 2005 25 11 2235 2237 10.1161/01.ATV.0000189303.45609.1f 16258147

[B13] de Valk B Marx JJ Iron, atherosclerosis, and ischemic heart disease Arch Intern Med 1999 159 14 1542 1548 10.1001/archinte.159.14.1542 10421276

[B14] Gillum RF Body iron stores and atherosclerosis Circulation 1997 96 10 3261 3263 9396411

[B15] Rossi E McQuillan BM Hung J Thompson PL Kuek C Beilby JP Serum ferritin and C282Y mutation of the hemochromatosis gene as predictors of asymptomatic carotid atherosclerosis in a community population Stroke 2000 31 12 3015 3020 11108765 10.1161/01.str.31.12.3015

[B16] Haidari M Javadi E Sanati A Hajilooi M Ghanbili J Association of increased ferritin with premature coronary stenosis in men Clin Chem 2001 47 9 1666 1672 11514401

[B17] Manfroi WC Zago AJ Caramori PR Cruz R Oliveira J Kirschnick LS Ordovas K Candiago RH de Souza J Ribeiro LW Does serum ferritin correlate with coronary angiography findings? Int J Cardiol 1999 69 2 149 153 10.1016/S0167-5273(99)00020-0 10549838

[B18] Ooi GC Khong PL Chan GC Chan KN Chan KL Lam W Ng I Ha SY Magnetic resonance screening of iron status in transfusion-dependent beta-thalassaemia patients Br J Haematol 2004 124 3 385 390 10.1046/j.1365-2141.2003.04772.x 14717788

[B19] Gandon Y Olivie D Guyader D Aube C Oberti F Sebille V Deugnier Y Non-invasive assessment of hepatic iron stores by MRI Lancet 2004 363 9406 357 362 10.1016/S0140-6736(04)15436-6 15070565

[B20] Rose C Vandevenne P Bourgeois E Cambier N Ernst O Liver iron content assessment by routine and simple magnetic resonance imaging procedure in highly transfused patients Eur J Haematol 2006 77 2 145 149 10.1111/j.0902-4441.2006.t01-1-EJH2571.x 16608501

[B21] Anderson LJ Holden S Davis B Prescott E Charrier CC Bunce NH Firmin DN Wonke B Porter J Walker JM Cardiovascular T2-star (T2*) magnetic resonance for the early diagnosis of myocardial iron overload Eur Heart J 2001 22 23 2171 2179 10.1053/euhj.2001.2822 11913479

[B22] Meloni A Ramazzotti A Positano V Salvatori C Mangione M Marcheschi P Favilli B De Marchi D Prato S Pepe A Evaluation of a web-based network for reproducible T2* MRI assessment of iron overload in thalassemia Int J Med Inform 2009 78 8 503 512 10.1016/j.ijmedinf.2009.02.011 19345609

[B23] Di Tucci AA Matta G Deplano S Gabbas A Depau C Derudas D Caocci G Agus A Angelucci E Myocardial iron overload assessment by T2* magnetic resonance imaging in adult transfusion dependent patients with acquired anemias Haematologica 2008 93 9 1385 1388 10.3324/haematol.12759 18603557

[B24] McLeod C Fleeman N Kirkham J Bagust A Boland A Chu P Dickson R Dundar Y Greenhalgh J Modell B Deferasirox for the treatment of iron overload associated with regular blood transfusions (transfusional haemosiderosis) in patients suffering with chronic anaemia: a systematic review and economic evaluation Health Technol Assess 2009 13 1 iii iv ix-xi, 1-121 19068191 10.3310/hta13010

[B25] O'Leary DH Polak JF Kronmal RA Manolio TA Burke GL Wolfson SK Jr Carotid-artery intima and media thickness as a risk factor for myocardial infarction and stroke in older adults. Cardiovascular Health Study Collaborative Research Group N Engl J Med 1999 340 1 14 22 9878640 10.1056/NEJM199901073400103

[B26] Ascherio A Rimm EB Giovannucci E Willett WC Stampfer MJ Blood donations and risk of coronary heart disease in men Circulation 2001 103 1 52 57 11136685 10.1161/01.cir.103.1.52

[B27] Ramakrishna G Rooke TW Cooper LT Iron and peripheral arterial disease: revisiting the iron hypothesis in a different light Vasc Med 2003 8 3 203 210 10.1191/1358863x03vm493ra 14989563

[B28] Ross R The pathogenesis of atherosclerosis: a perspective for the 1990s Nature 1993 362 6423 801 809 10.1038/362801a0 8479518

[B29] Kablak-Ziembicka A Przewlocki T Tracz W Pieniazek P Musialek P Sokolowski A Gender differences in carotid intima-media thickness in patients with suspected coronary artery disease Am J Cardiol 2005 96 9 1217 1222 10.1016/j.amjcard.2005.06.059 16253585

[B30] Steinberg D Parthasarathy S Carew TE Khoo JC Witztum JL Beyond cholesterol. Modifications of low-density lipoprotein that increase its atherogenicity N Engl J Med 1989 320 14 915 924 10.1056/NEJM198904063201407 2648148

[B31] Steinberg D Antioxidants and atherosclerosis. A current assessment Circulation 1991 84 3 1420 1425 1884464 10.1161/01.cir.84.3.1420

[B32] Steinbrecher UP Zhang HF Lougheed M Role of oxidatively modified LDL in atherosclerosis Free Radic Biol Med 1990 9 2 155 168 10.1016/0891-5849(90)90119-4 2227530

[B33] Halliwell B Chirico S Lipid peroxidation: its mechanism, measurement, and significance Am J Clin Nutr 1993 57 5 Suppl 715S 724S discussion 724S-725S 8475889 10.1093/ajcn/57.5.715S

[B34] Chrysohoou C Panagiotakos DB Pitsavos C Kosma K Barbetseas J Karagiorga M Ladis I Stefanadis C Distribution of serum lipids and lipoproteins in patients with beta thalassaemia major; an epidemiological study in young adults from Greece Lipids Health Dis 2004 3 3 10.1186/1476-511X-3-3 15023232 PMC385250

[B35] Cheung YF Chow PC Chan GC Ha SY Carotid intima-media thickness is increased and related to arterial stiffening in patients with beta-thalassaemia major Br J Haematol 2006 135 5 732 734 10.1111/j.1365-2141.2006.06349.x 17107355

